# Push-off asymmetry and kinetic strategies in functional ankle instability during continuous vertical jump tasks

**DOI:** 10.3389/fbioe.2026.1772532

**Published:** 2026-02-10

**Authors:** Mingjie Lin, Zilong Wang, Jie Lu, Weibo Shao, Lingyue Meng, Qiuxia Zhang, Xiangdong Wang

**Affiliations:** 1 School of Physical Education, Jimei University, Xiamen, China; 2 The Second Affiliated Hospital of Nanjing University of Chinese Medicine, Nanjing, China; 3 School of Physical Education, Soochow University, Suzhou, China

**Keywords:** asymmetry, continuous vertical jump, functional ankle instability, linear mixed model, power decline, push-off kinetics

## Abstract

**Objective:**

To investigate the kinetic performance, power decline trajectory, and lower limb load asymmetry characteristics during the push-off phase of a 30-repetition continuous vertical jump task in individuals with functional ankle instability (FAI), and to reveal potential motor control deviations under cyclic high-output conditions.

**Methods:**

Eleven males with unilateral FAI and eleven healthy control participants were recruited to complete a task consisting of 30 continuous double-leg vertical jumps. Kistler force plates and Visual3D software were utilized to extract relevant kinetic data and corresponding asymmetry indices. A linear mixed model (LMM) was constructed based on jump order to analyze kinetic trajectories. Furthermore, the rate of power decline was quantified via individual regression slopes and compared between groups.

**Results:**

(1) The FAI group exhibited significantly higher asymmetry in peak vertical ground reaction force (vGRF) (*p* = 0.030). (2) While no significant Group × Jump Order interaction was observed for overall jump height or impulse (*p* > 0.05), the FAI group presented a significantly flatter decline slope in peak vGRF compared to controls (*p* = 0.046), indicating a distinct output strategy rather than a general performance failure.

**Conclusion:**

Although individuals with FAI are capable of maintaining overall power output levels comparable to healthy controls during cyclic high-output tasks, they exhibit an unbalanced load distribution and a “low amplitude–low decline” kinetic output mode. This reflects a compensatory motor control structure formed over the long term. Bilateral load asymmetry and the rhythm of output decline are more indicative of potential control deficits than overall performance metrics. Future assessment and rehabilitation should prioritize the restoration of strength, neuromuscular control, and kinetic balance on the affected side to improve dynamic stability under fatigue conditions and reduce the risk of re-injury.

## 1 Introduction

Ankle sprains represent one of the lower limb injuries with the highest incidence rates in competitive sports and daily physical activities, accounting for 16%–40% of all sports injuries (Doherty et al., 2014; Herzog et al., 2019; Jungmann et al., 2023; Tyler et al., 2025). Although the majority of individuals achieve short-term relief from clinical symptoms following an acute sprain, substantial research indicates that approximately 30%–70% of patients continue to experience varying degrees of ankle instability, recurrent sprains, or functional decline for months or even years post-injury, potentially progressing to functional ankle instability (FAI) (Sonsukong et al., 2024; Lin et al., 2022; Wang et al., 2024a). FAI is typically characterized by a subjective sensation of the ankle “giving way,” a history of recurrent sprains, and diminished motor control capabilities during dynamic tasks, accompanied by deep-seated functional deficits such as reduced proprioception, delayed muscle reaction time, and neuromuscular control insufficiency (Hertel and Corbett, 2019; Simpson et al., 2019; Labanca et al., 2021). These persistent functional impairments not only increase the risk of re-injury but may also lead to long-term compensations in gait and movement patterns, subsequently affecting the mechanical load distribution of lower limb joints and resulting in a decline in daily activity capacity and quality of life (Wang et al., 2026).

The vertical jump is one of the most typical and fundamental forms of lower limb explosive power output. It is widely employed in physical fitness training and functional assessment, as well as in sports requiring frequent jumping and landing, such as basketball, volleyball, and badminton (Markovic, 2007; Ziv and Lidor, 2010; Sattler et al., 2012). Compared to single vertical jump tasks, the continuous vertical jump task—which involves repeatedly completing high-intensity take-offs and landings within a short timeframe—more closely approximates cyclic high-output scenarios in actual competition (e.g., continuous jumping, rebounding, and blocking). Consequently, it is more effective at inducing neuromuscular fatigue and amplifying potential control deficits within a brief period (Wang et al., 2024a). Specifically, the peak vertical ground reaction force (vGRF) and impulse during the push-off phase reflect the neuromuscular system’s explosive drive and mechanical load tolerance, while their decay trajectories (Slope) under repetitive tasks serve as sensitive indicators of neuromuscular fatigue resistance. Furthermore, the Asymmetry Index (AI) reveals the compensatory load redistribution strategy between the affected and healthy limbs. Existing studies utilizing electromyography and kinematics have demonstrated that individuals with FAI exhibit differences in recruitment patterns, synergistic control, and pre-activation modes of ankle musculature compared to healthy populations during single jump or landing tasks, accompanied by compromised dynamic postural control (Wang et al., 2024a; Feger et al., 2014). Research has also reported that individuals with FAI demonstrate altered landing postures, insufficient shock absorption capabilities, and joint load asymmetry during the landing phase (Zhong et al., 2024; Lin et al., 2019). Current single-repetition assessments often fail to capture the cumulative neuromuscular fatigue that precipitates ‘giving way’ episodes in real-world sports. Therefore, investigating kinetic alterations under cyclic high-output conditions is crucial for identifying latent control deficits. However, there is currently a lack of systematic evidence regarding the performance of individuals with FAI during cyclic high-output tasks such as the continuous vertical jump. In particular, it remains unclear whether individuals with FAI can maintain stable push-off kinetics during continuous high-intensity jumping, and how their power decline trajectories and load allocation strategies evolve. These issues are directly relevant to the formulation of exercise prescriptions, functional assessment, and return-to-sport decision-making.

Lower limb asymmetry serves as a critical dimension for evaluating the balance of function and load allocation between the lower extremities (Cone and Lee, 2021). The AI, constructed based on differences between bilateral indicators, is widely used to quantify left-right discrepancies in metrics such as jump height, ground reaction force, and impulse (Zhong et al., 2024; Lin et al., 2019). While moderate asymmetry may be functional in certain specialized technical movements—such as the dominant leg undertaking more of the take-off task—excessive or persistent asymmetry is often associated with decreased athletic performance, increased injury risk, and compensatory movement patterns, particularly under conditions of accumulated fatigue or high-frequency repetitive action (Helme et al., 2021; Fort-Vanmeerhaeghe et al., 2020; Sun et al., 2025). For individuals with FAI, characterized primarily by unilateral ankle functional impairment, systemic alterations in lower limb load allocation strategies are highly probable. On one hand, the injured ankle may reduce its load due to protective strategies; on the other hand, the healthy lower limb may passively assume greater impact and propulsion tasks, thereby exacerbating bilateral asymmetry (Doherty et al., 2015; Delahunt et al., 2006). Previous studies concerning individuals with FAI have largely focused on asymmetry differences during single landing or jumping tasks, whereas actions in actual sports are often continuous and repetitive. Under the high-frequency cyclic task of continuous vertical jump, it has not yet been clarified whether differences exist between individuals with FAI and healthy populations regarding kinetic output and load asymmetry evolution during the push-off phase, nor whether such differences accumulate or alter as the jump order progresses.

In view of this, the present study utilizes the continuous vertical jump as a context to compare differences in push-off kinetics and lower limb load asymmetry between individuals with FAI and healthy controls, with a specific focus on kinetic decline characteristics as the jump order progresses. The aim is to reveal potential kinetic limitations of individuals with FAI during cyclic high-output tasks, providing a basis for functional assessment and training protocols. This study proposes the following hypotheses: 1) Individuals with FAI would exhibit altered decline trajectories in kinetic output and load asymmetry compared to healthy controls under fatigue conditions; 2) Individuals with FAI will exhibit differences in push-off kinetic performance and load allocation modes compared to healthy controls.

## 2 Methods

### 2.1 Participants

The sample size was estimated using G*Power 3.1.9 software. Given that G*Power is based on ANOVA assumptions, the *F*-test family (ANOVA: Repeated measures, within-between interaction) was utilized as a proxy for the LMM analysis. The specific parameters were set as follows: effect size Cohen’s *f* = 0.40. This value was conservatively selected based on our previous research (Wang et al., 2024b; Wang et al., 2024c), which reported interaction effects (*η* ^*2*^) ranging from 0.128 to 0.283. We chose *f* = 0.40 (equivalent to *η* ^*2*^ ≈ 0.14) to align with the lower bound of this range, ensuring sensitivity to smaller effect sizes. Type I error probability α = 0.05, Power (1−*β*) = 0.80, number of groups = 2, and number of measurements = 2. Although the experimental task involved 30 consecutive jumps, we conservatively set this parameter to 2 to model the critical contrast (e.g., initial vs. final jumps). This conservative approach avoids the overestimation of statistical power associated with high-frequency repeated measures inputs in G*Power. A conservative correlation among repeated measures of 0.5 and a non-sphericity correction *ϵ* = 1 were applied. The calculated minimum sample size was 16 individuals (8 per group). Considering a potential invalid sample rate of approximately 20%, this study ultimately screened 11 males with unilateral FAI as the experimental group, based on the screening criteria of the International Ankle Consortium (Gribble et al., 2014). Specifically, the Cumberland Ankle Instability Tool (CAIT) was selected as the primary patient-reported outcome measure (PROM). This selection aligns with recent recommendations emphasizing the importance of identifiability and widespread adoption in PROM selection for Chronic Ankle Instability (CAI) (Luan et al., 2025a). Furthermore, clinical examinations were performed to assess ligamentous laxity. Notably, the anterior drawer test was utilized as an exclusion tool; consistent with its documented diagnostic value and criterion validity (Luan et al., 2025b), only participants with negative results were included to exclude Mechanical Ankle Instability (MAI) and ensure a homogeneous FAI sample. Simultaneously, 11 participants with no history of ankle injury were matched from a pool of healthy individuals to form the control group, based on the morphological characteristics and athletic background of the FAI group. Given that 92% of ankle sprains occur on the dominant side (Beynnon et al., 2002), individuals with FAI on the dominant side were selected, and matched with the dominant side of the control group. The dominant and non-dominant sides for both groups were determined via the ball-kicking test (Ren et al., 2022). All assessment and screening procedures were conducted by a rehabilitation therapist with extensive clinical experience. All participants were fully informed of the testing process and signed informed consent forms prior to the experiment. The study was approved by the Ethics Committee of Soochow University (Ethics No.: SUDA20241209H14). Basic information of the participants is presented in Table 1.

**TABLE 1:** Participant characteristics (M ± SD).

Characteristic	FAI group (*n* = 11)	Control group (*n* = 11)
Age (year)	22.6 ± 1.1	22.3 ± 2.0
Height (cm)	178.3 ± 8.0	178.7 ± 6.0
Weight (kg)	74.2 ± 9.7	75.6 ± 7.9
BMI (kg/m^2^)	23.3 ± 2.0	23.2 ± 1.5
CAIT score	17.5 ± 2.2	29.8 ± 0.4

*p < 0.05, similarly hereinafter.

The inclusion and exclusion criteria for the FAI and control groups, combined with previous research (Wang et al., 2024a; Wang et al., 2024b; Meng et al., 2022; Meng et al., 2025), were as follows:

FAI Group Inclusion Criteria: ① A history of at least one significant unilateral ankle sprain, with the initial sprain occurring at least 12 months prior to the trial to ensure the condition had progressed to a chronic stage and to exclude acute physiological interference, accompanied by inflammatory symptoms (pain, swelling, etc.) and resulting in at least 1 day of interrupted physical activity; ② A history of “giving way,” recurrent sprains, and/or feelings of instability in the ankle; ③ CAIT score ≤24; ④ FAI present in only one ankle.

FAI Group Exclusion Criteria: ① History of acute ankle sprains, surgery, or fractures in the lower limbs; ② Acute injury to musculoskeletal structures of other lower limb joints within 1 month prior to the formal trial; ③ Bilateral FAI; ④ Congenital joint deformities; ⑤ Positive results for talar tilt or anterior drawer tests (indicating mechanical instability).

Control Group Inclusion Criteria: ① CAIT score ≥28; ② Matched morphological characteristics and athletic background with the FAI group.

Control Group Exclusion Criteria: ① History of lower limb surgery; ② Acute joint injury within 1 month prior to the trial; ③ Inability to complete assessments as required or missing data during the trial.

### 2.2 Experimental design and procedure

All participants in this study were required to complete a task consisting of 30 continuous vertical jumps. Prior to the start of the experiment, participants wore tight-fitting shorts and athletic shoes provided uniformly by the laboratory.

### 2.3 Data collection and analysis

All data were collected using Kistler 3D force plates (90 cm × times × 60 cm × 10 cm, Model: 9287B, Switzerland, 1,000 Hz). Visual3D (C-Motion Inc., USA) was utilized for kinetic analysis of the collected raw data. A fourth-order low-pass Butterworth filter was employed to smooth the force plate data (50 Hz). Lower limb joint angles were calculated using the Euler angle method. The instants of take-off and touchdown were determined based on the vGRF crossing a threshold of 10 N (Cui et al., 2025). Given that this study focuses on the explosive force generation mechanism of the vertical jump, all kinetic metrics were extracted from the push-off phase. The push-off phase was defined as the period from the earliest moment the vGRF passed the local minimum (completion of cushioning) during the contact phase (onset of push-off) to the instant the vGRF dropped below 10 N at take-off (end of push-off).

Based on the kinetic curves of each complete push-off phase within the 30 continuous vertical jumps, the following variables were extracted:

1. Jump Height: Calculated based on flight time using the ballistic Formula 1, unit (cm), where h is jump height, g is gravitational acceleration, and T is flight time:
$$h=gT^{2}/8$$ (1)
2. Push-off Phase vGRF Variables: ① Peak vGRF during push-off: The maximum value of vGRF within the push-off phase, unit (N); ② Time to Peak vGRF (T_vGRF): Time from the onset of push-off to peak vGRF, unit (ms); ③ Push-off Vertical Impulse (Imp): The integral of the vGRF–time curve during the push-off phase (Formula 2), unit (N·s), where Fz_(t)_ is the instantaneous value of vGRF, t_onset_ is the start time of the push-off phase, t_to_ is the instant of take-off, and dt is the time differential.
$$\text{Im}p_{\text{prop}}=\left.\int_{t_{\text{onset}}}^{t_{\text{to}}}\right|F_{z}\left(t\right)|\text{dt}$$ (2)
3. Lower Limb AI: To analyze the load allocation strategy of participants with FAI during the push-off phase, the asymmetry of peak force and impulse for the left and right feet was calculated respectively: ① Peak vGRF AI (Formula 3); ② Impulse AI (Imp AI, Formula 4). Higher values indicate a more uneven distribution of load between the lower limbs. The absolute value was calculated to assess the magnitude of asymmetry.
$${\text{AI}}_{\text{Peak}\;\text{vGR}F}=\frac{\left|{\text{vGR}F}_{L}‐{\text{vGR}F}_{R}\right|}{{\text{vGR}F}_{L}+{\text{vGR}F}_{R}}\times 100\%$$ (3)
$${\text{AI}}_{I\text{mp}}=\frac{\left|{\text{Imp}}_{L}‐{\text{Imp}}_{R}\right|}{{\text{Imp}}_{L}+{\text{Imp}}_{R}}\times 100\%$$ (4)
4. Based on the 30 continuous vertical jumps, with jump order as the continuous independent variable, the trajectory of each kinetic variable during the continuous jumping process was analyzed. Furthermore, the regression slope for each participant was calculated to quantify the rate of decline in explosive power, change in asymmetry, and neuromuscular output decay during fatigue.

### 2.4 Statistical analysis

Statistical analyses were performed using SPSS 27. Continuous variables were first tested for normality using the Shapiro–Wilk test. A Linear Mixed Model (LMM) was constructed, with Group (FAI and Control), Jump Order (1–30), and their interaction set as fixed effects, and participant ID set as a random intercept. To address the time dependency of the continuous vertical jump sequence, structure was modeled using a First-order Autoregressive Model (AR(1)), which is appropriate for longitudinal data where correlations between repeated measures decay with increasing time lag. Model assumptions, including the normality of residuals, were verified visually using Q-Q plots. When fixed effects reached the pre-set significance level, Bonferroni corrections were applied for post-hoc comparisons. Additionally, to quantify the individual rate of fatigue decline, linear regression models were fitted for kinetic indicators against jump order at the individual level to extract the regression slope for each participant. Independent sample t-tests were used to compare group differences in slopes. The significance level for two-tailed tests was set at *α* = 0.05.

## 3 Results

### 3.1 Trends of jump performance and kinetic variables

**FIGURE 1:**
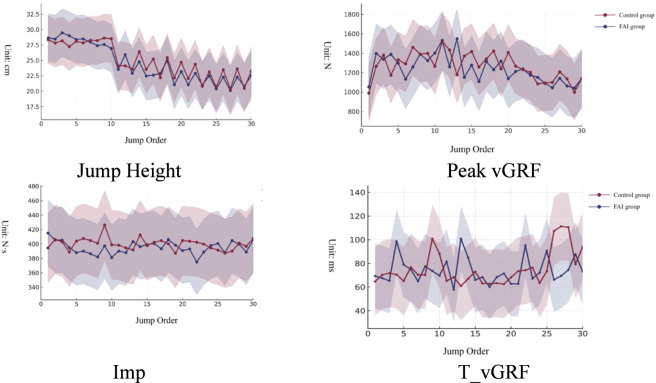
Trends of jump performance and push-off phase kinetic variables during 30 continuous vertical jumps.

Regarding jump height, the Linear Mixed Model results indicated a statistically significant Main Effect for Jump Order (*F* = 2.880, *p* < 0.001), suggesting that jump height undergoes systematic changes with the increase in continuous jump repetitions. The Main Effect for Group was not statistically significant (*p* > 0.05), indicating no significant difference in overall jump height between the FAI group and the control group. Furthermore, the Group × Jump Order Interaction Effect was not statistically significant (*p* > 0.05), suggesting similar trajectories of change in jump height across continuous jumps for both groups. Marginal means results further revealed that both groups maintained greater jump heights during the first 1–10 jumps, followed by a declining trend as the jump order increased. No statistical differences were observed in push-off Peak vGRF, T_vGRF, or Imp variables (*p* > 0.05) (Figure 1).

### 3.2 AI variables

**FIGURE 2:**
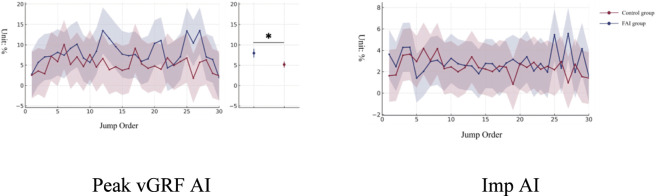
Evolution characteristics of inter-limb load AI during 30 continuous vertical jumps.

A Main Effect for Group was observed for the Peak vGRF AI variable (*F* = 4.914, *p* = 0.030), indicating a difference in the bilateral symmetry of peak force allocation during the push-off phase between the FAI group and the control group. Marginal estimated means showed that the Peak vGRF AI of the FAI group was higher than that of the control group (FAI group: 7.946% ± 0.866%; Control group: 5.153% ± 0.915%), demonstrating greater asymmetry. Neither the Main Effect for Jump Order nor the Group × Jump Order Interaction Effect reached significance (*p* > 0.05) (Figure 2).

### 3.3 Slope variables during continuous vertical jumps

**FIGURE 3:**
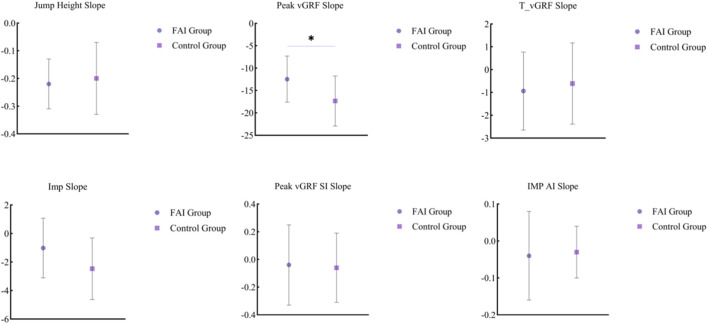
Comparison of regression slopes of kinetic variables across 30 continuous vertical jumps.

A statistical difference between the two groups was observed for the vGRF Peak Slope variable (*t* = 2.127, *p* = 0.046). The magnitude of the decline in the vGRF Peak Slope for the FAI group was smaller than that of the control group (Figure 3).

## 4 Discussion

This study represents a novel attempt to shift the focus from “discrete outcome assessment” to “continuous process monitoring” in FAI research. By utilizing a linear mixed model and individual regression slope analysis based on a 30-repetition continuous vertical jump task, we aimed to quantify the dynamic decay trajectories of push-off kinetics and the evolution of load asymmetry in individuals with FAI under cyclic high-output conditions. Unlike traditional single vertical jumps, discrete landing tests, or static balance assessments, the continuous vertical jump paradigm combined with slope analysis more closely approximates the repetitive take-off and landing scenarios in real sports, thereby offering greater sensitivity in capturing potential neuromuscular regulation strategies in individuals with FAI during high-frequency, rapid-transition movements.

Regarding the overall results, no interaction effects were observed in key push-off kinetic variables such as jump height, vGRF Peak, T_vGRF, and Impulse. While this contrasts with our initial expectation (Hypothesis 1) of a rapid performance decrement in FAI, it provides novel insight into their motor control strategy. It appears that individuals with FAI prioritize the maintenance of global output levels (jump height) through compensatory mechanisms, rather than succumbing to immediate mechanical failure. Furthermore, due to prior ankle injury, individuals with FAI may experience varying degrees of impairment in local stability, proprioception, and force control. Consequently, they may rely more on the synergy and compensation of other lower limb joints to offset the insufficient contribution of the affected side during high-output tasks (Ono et al., 2023; Kim et al., 2018), thereby maintaining output levels similar to the healthy group in the short term. Both groups maintained high performance during the first 1–10 jumps, followed by a consistent declining trend. This “fatigue–performance decline” mode aligns with the findings of Schmitz et al. (2014), who observed gradual changes in lower limb landing mechanics during intermittent exercise, and is consistent with the conclusion of Fort-Vanmeerhaeghe et al. (2020) in young competitive athletes, which stated that overall performance changes in explosive movements during early fatigue stages are limited, whereas local indicators and asymmetry are more sensitive. This also echoes the view of Alba-Jiménez et al. (2022) in a review on neuromuscular fatigue monitoring in team sports, namely, that functional performance differences prior to fatigue are often indistinct, while deeper control discrepancies are more easily revealed in local parameters or long-term compensatory patterns.

Although the overall kinetic trajectory did not reflect an interaction effect, a Main Effect for Group was observed in the vGRF Peak AI and vGRF Peak Slope variables. The FAI group exhibited higher vGRF Peak AI, indicating more unbalanced left-right load allocation during the push-off phase. This is consistent with Hypothesis 2 of this study, stating that individuals with FAI differ from healthy populations in load allocation modes. This aligns with the conclusion of Sonsukong et al. (2024) regarding direction-dependent lateral control in ankle kinematics and electromyography of CAI individuals during multi-directional single-leg landing tasks, and coincides with the systematic review by Simpson et al. (2019), which pointed out that CAI individuals exhibit impaired dynamic postural stability and abnormal landing kinetics during unilateral landing tasks. A systematic review and meta-analysis by Jeon et al. (2021) further confirmed structural changes in muscle activation and mechanical control in populations with ankle instability, suggesting a reconstruction of their motor control strategies. The higher vGRF Peak AI observed in this study is highly consistent with the aforementioned conclusions, supporting the notion that individuals with FAI rely more on the healthy side to undertake propulsion tasks during high-output tasks.

Notably, this study also observed that the FAI group presented a flatter declining trend in the vGRF Peak Slope variable, with a significantly smaller decline magnitude compared to the healthy population. This large effect size underscores a distinct difference in fatigue response. Superficially, this seems to imply that individuals with FAI experience slower fatigue decline during repetitive jump tasks; however, when combined with their higher asymmetry, this may suggest a potential “low amplitude-low decline” stabilized output strategy. Although speculative due to the absence of neuromuscular data in this study, it is possible that individuals with FAI adopt a more conservative output mode with smaller fluctuations from the onset of the task to reduce mechanical exposure of the affected joint and maintain overall stability. This leaves less room for performance decline, resulting in a smaller slope magnitude. However, no group differences were observed in overall push-off vGRF Peak and Impulse variables, implying that the lower output of individuals with FAI does not directly reflect a reduction in overall peak force or impulse. The reason lies in the fact that the lower output of individuals with FAI does not occur at the gross level, but rather at the local level where the contribution of the affected side is reduced; concurrently, the compensatory contribution of the healthy side offsets the deficiency in the overall peak, thereby masking differences at the task level (Kong et al., 2023; Dayakidis and Boudolos, 2006; Ono et al., 2023; Kim et al., 2018). Therefore, this strategic “low amplitude–low decline” mode is difficult to identify relying solely on overall peak or overall impulse. It reflects a profound remodeling of the motor control system: the central nervous system prioritizes the “safety” of the injured joint by constraining its output variability, thereby “freezing” the available degrees of freedom. This mechanism requires more sensitive local kinetic indicators such as bilateral load allocation to be accurately captured. In other words, this “low amplitude–low decline” pattern found in this study likely stems from long-term adaptations in the organization of force output in individuals with FAI (Dejong et al., 2020), rather than an absolute output insufficiency. This adaptive compensation, characterized by “maintenance of overall output—deviation of local contribution,” is a key mechanistic feature of FAI in dynamic tasks.

In contrast, variables that did not show group differences, such as T_vGRF, Impulse, and jump height, may reflect consistency between the two groups in overall explosive rhythm, joint extension timing, and power integration efficiency. Previous studies have also noted that in comprehensive indicators like jump height, differences between FAI and healthy groups are often less sensitive than local indicators such as landing control and load asymmetry (Wang et al., 2024a). Therefore, the phenomenon in this study where some indicators show differences while others remain consistent suggests that kinetic deficits in FAI possess a certain selectivity, more likely concentrated in load allocation strategies and local output regulation rather than overall output capacity itself. This inference is supported by electromyographic evidence suggesting that individuals with ankle instability exhibit altered muscle recruitment patterns and multi-joint coordination deficits (Labanca et al., 2021). These neuromuscular adaptations likely function as compensatory mechanisms to maintain task completion despite local functional impairments, rather than manifesting directly as a decline in overall explosive power.

In summary, individuals with FAI exhibit kinetic characteristics of “overall output maintenance, load allocation deviation, and unique decline rhythm” during cyclic high-output tasks. Although their explosive power levels are similar to those of healthy populations, they rely on compensatory load allocation and output rhythm adjustments to make up for control deficits on the affected side, reflecting stable yet long-term motor control deviations. Clinically, this suggests that practitioners should not solely rely on macroscopic metrics like jump height to assess recovery, as these may mask underlying deficits due to compensatory strategies. Instead, training and rehabilitation should focus on restoring the symmetry of kinetic outputs and the stability of force decay rates. Improving lateral reliance by strengthening force, neuromuscular control, and lower limb synergy on the affected side is essential to disrupt the “low amplitude–low decline” compensatory loop. Simultaneously, incorporating high-repetition or continuous jumping training aids in improving control capabilities under fatigue, providing a more targeted basis for return-to-sport assessment and injury prevention.

This study has certain limitations. 1) Although the sample size (n = 11 per group) exceeded the requirement of the *a priori* power analysis (n = 8 per group), the estimation was based on RM-ANOVA assumptions (G*Power), which serves as an approximation and may not fully capture the complexity of the LMM structure (e.g., random slopes). While the 37.5% sample surplus helps mitigate this, the study may still have limited statistical power to detect smaller Group × Jump Order interaction effects. Future studies with larger cohorts and simulation-based power analyses are recommended to verify these findings; 2) The participants were all male, which may limit the generalization of the results to populations of different sexes, ages, and training backgrounds; 3) Although 30 continuous vertical jumps can induce initial fatigue, it may still be insufficient to expose deeper motor control differences. Additionally, only one trial of 30 jumps was performed to avoid excessive cumulative fatigue that might alter natural movement strategies, which limits the assessment of between-trial reliability; 4) Electromyographic or central control indicators were not synchronized during collection, thus preventing a comprehensive explanation of the neuromuscular mechanisms behind the compensatory strategies. Future research should combine multimodal data such as EMG, joint synergy, and neural drive to help construct a more complete FAI control model.

## 5 Conclusion

This study identified distinct kinetic adaptations in individuals with FAI during a 30-repetition continuous vertical jump task. While overall power output trajectories remained similar to healthy controls, the FAI group exhibited significantly higher peak force asymmetry and a flatter output decline slope. These findings indicate a “maintenance of output–deviation of load” strategy. Clinically, this suggests that macroscopic performance metrics may mask underlying deficits. Consequently, rehabilitation and return-to-sport assessments should prioritize monitoring bilateral load allocation and fatigue-induced kinetic changes to effectively target neuromuscular control deficits and reduce re-injury risks.

## Data Availability

The raw data supporting the conclusions of this article will be made available by the authors, without undue reservation.
